# Using HIV Networks to Inform Real Time Prevention Interventions

**DOI:** 10.1371/journal.pone.0098443

**Published:** 2014-06-05

**Authors:** Susan J. Little, Sergei L. Kosakovsky Pond, Christy M. Anderson, Jason A. Young, Joel O. Wertheim, Sanjay R. Mehta, Susanne May, Davey M. Smith

**Affiliations:** 1 Department of Medicine, University of California, San Diego, La Jolla, California, United States of America; 2 Department of Biostatistics, University of Washington, Seattle, Washington, United States of America; 3 Veterans Affairs San Diego Healthcare System, San Diego, California, United States of America; University of British Columbia, Canada

## Abstract

**Objective:**

To reconstruct the local HIV-1 transmission network from 1996 to 2011 and use network data to evaluate and guide efforts to interrupt transmission.

**Design:**

HIV-1 *pol* sequence data were analyzed to infer the local transmission network.

**Methods:**

We analyzed HIV-1 *pol* sequence data to infer a partial local transmission network among 478 recently HIV-1 infected persons and 170 of their sexual and social contacts in San Diego, California. A transmission network score (TNS) was developed to estimate the risk of HIV transmission from a newly diagnosed individual to a new partner and target prevention interventions.

**Results:**

HIV-1 *pol* sequences from 339 individuals (52.3%) were highly similar to sequences from at least one other participant (i.e., clustered). A high TNS (top 25%) was significantly correlated with baseline risk behaviors (number of unique sexual partners and insertive unprotected anal intercourse (p = 0.014 and p = 0.0455, respectively) and predicted risk of transmission (p<0.0001). Retrospective analysis of antiretroviral therapy (ART) use, and simulations of ART targeted to individuals with the highest TNS, showed significantly reduced network level HIV transmission (p<0.05).

**Conclusions:**

Sequence data from an HIV-1 screening program focused on recently infected persons and their social and sexual contacts enabled the characterization of a highly connected transmission network. The network-based risk score (TNS) was highly correlated with transmission risk behaviors and outcomes, and can be used identify and target effective prevention interventions, like ART, to those at a greater risk for HIV-1 transmission.

## Introduction

Communicable diseases spread through contacts within social or sexual networks [1], [2]. The dynamic structure of these networks govern the spread of the infection [3], and can inform public health measures to contain infectious epidemics [4]. A common way to define important network features is through interview and partner tracing [5], but these techniques are of limited value when the infectious disease has a long incubation period between transmission and disease state and a low transmission rate per contact, like human immunodeficiency virus (HIV-1) [6]. Recent advances in molecular epidemiology have greatly enhanced our ability to characterize transmission networks of infectious diseases [7].

The high evolutionary rate of HIV-1 gives rise to an essentially unique HIV-1 genetic sequence for each infected individual, enabling detailed studies of local and global epidemics[8], [9]. Because partial HIV-1 *pol* sequences are generated for routine drug resistance testing [10], the data necessary to perform such molecular analyses are often readily available and centralized in commercial laboratories. These laboratories interpret HIV sequence data to estimate antiretroviral drug resistance [11]. Without the immediate prospect of a broadly effective vaccine for HIV-1, molecular epidemiology has the potential to identify individuals most likely to transmit infection, who could be targeted for efficient and effective delivery of scarce prevention resources.

In this study, we analyzed HIV-1 *pol* sequences generated over a period of more than 15 years from recently HIV-1 infected individuals and their sexual and social contacts identified in San Diego, California. Based on these data, we inferred the local molecular transmission network and evaluated if network hubs could be targeted for effective prevention efforts.

## Methods

### Study Population

HIV-1 screening was offered to adults and adolescents between 1996 and 2011 at multiple HIV-1 testing and counseling sites in San Diego, California [12]. All HIV-1-positive individuals were offered study participation with confidential partner services. All persons identified with recent infection who were antiretroviral treatment (ART)-naïve formed the San Diego Primary Infection Cohort (SDPIC). HIV-1 screening was also provided to recent sexual and social network contacts of newly infected participants. The UCSD Human Research Protections Program approved the study protocol, consent and procedures for consent. All study participants provided voluntary, written informed consent before any study procedures were undertaken.

An estimated date of infection (EDI) was computed for all recently infected participants as previously reported[13] (supplemental Table S1), that characterized acute HIV-1 infection in persons presenting with negative HIV-1/2 serologies and a positive HIV-1 RNA (Procleix HIV-1/HCV Assay: Chiron, Emeryville, California, and Genprobe, San Diego, California). HIV-1 risk behaviors (using computer assisted self-interviews), blood viral load (Amplicor, Roche) and CD4 count (flow cytometry) were obtained at baseline and every 12 weeks throughout follow-up. All participants were assessed for baseline HIV-1 transmitted drug resistance via bulk sequencing of the partial HIV-1 *pol* coding region (GeneSeq HIV-1; Monogram Biosciences, Inc., South San Francisco, CA or Viroseq v.2.0; Celera Diagnostics, Alameda, CA). The sequenced region included the protease gene and between 305 and 335 5′ amino-acids of the reverse transcriptase gene. Repeated HIV-1 *pol* sequences were generated in a subset of participants. The GenBank accession numbers for the 648 baseline *pol* sequences included in this analysis are KJ722809–KJ723456. In an effort to avoid unintended disclosure of study participants, data accompanying HIV sequences are limited to year of sampling, country of origin and a random unique participant ID. Although ART was not provided, treatment was generally encouraged.

### Transmission Network

**Figure 1:**
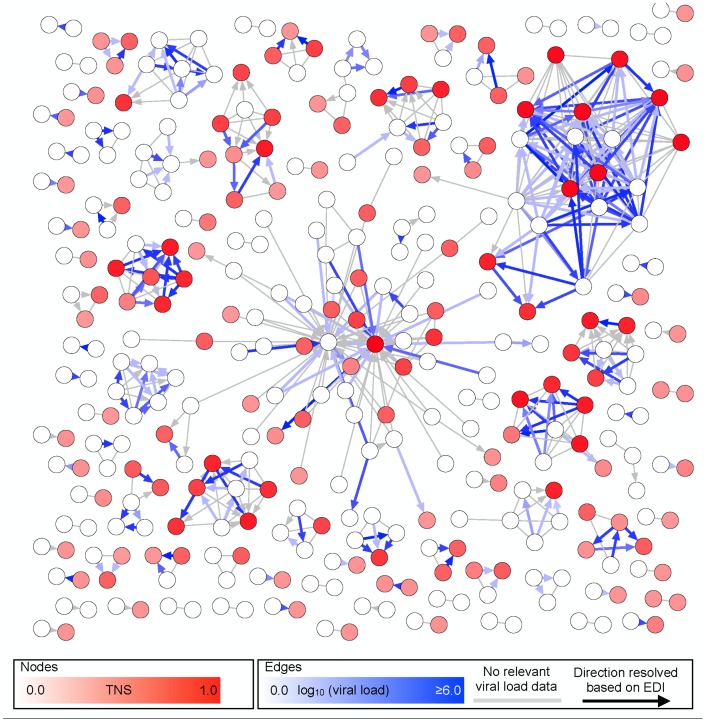
The inferred transmission network (excluding unconnected individuals) in the SDPIC. Only clustered individuals (nodes) within the network are shown (52.3%). Despite the likely presence of unsampled (i.e., missing) nodes, a partial HIV-1 transmission network is color coded; the intensity of coloring of nodes determined by their TNS score, while that for directed edges corresponds to the viral load of the putative initial partner at the timepoint closest to the transmission event. Absence of blue shading indicates that no VL was available for the sampled individual at any timepoint or that the direction of the edge could not be ascertained using EDI (see text). Absence of red shading indicates a TNS  = 0 (i.e. nodes that were unconnected at the time of enrollment). Nodes are connected with an edge (i.e., a line to indicate potential transmission) if the minimum distance between the respective *pol* sequences (i.e., possible transmission pairs) is less than 1.5%. A direction is assigned to an edge if the EDI for the secondary partner (i.e., putative “recipient”) is at least 30 days after the sampling date of the putative transmitting partner (i.e., putative “source”). The direction of transmission was resolved for in 332 of the 540 individuals (61.5%).

Sequence curation, alignment, and network inference were performed using either the HyPhy package [14] or freely available software (https://github.com/veg/HIV-1Clustering, https://github.com/veg/TN93, details and justification provided in Supplemental Methods). After quality control procedures to remove potential contaminant sequences [15], the partial transmission network was inferred based on the nucleotide genetic distances between bulk HIV-1 *pol* sequences from each participant (Figure 1) [16], [17]. In accordance with previous analyses [11], [18], [19], we linked two individuals (nodes) in the networks whenever their *pol* sequences were less than 1.5% distant (TN93 distance measure, see Figure S1 in File). The degree (connectivity) [20] of each individual was defined as the number of links (edges in the transmission network) to other individuals. Clusters were defined as connected components of the network comprising two or more nodes (Figure 1). Epidemiologic contact information was not a requirement for clustering in the molecular network, since the presence of a link does not imply direct transmission (but rather two recently related viruses). Whenever possible, we assigned a direction to the network edge (i.e., an arrow to indicate the likely transmission direction), if the EDI of the secondary partner (i.e., putative “recipient”) node was at least 30 days past the date at which the initial partner (i.e., putative “source”) sequence was isolated (Figure S2 in File S1). We conservatively assumed that chronically infected subjects had an EDI of at least 180 days from enrollment (based on the reliability of available detuned HIV assays to estimate the duration of recent infection [21]–[23]). Since multiple bulk HIV-1 *pol* sequences generated from participants with longitudinal follow-up were available and could boost our power to detect transmissions originating from chronically infected individuals, we used all available sequences to define links.

### Network Properties and Transmission Network Score

In order to determine whether baseline network characteristics could predict an individual's future transmission risk, we formulated a numeric transmission network score (TNS). Using only baseline data, we characterized the risk of HIV-1 transmission within the first year after study entry, for participants entering the study between 2005–2010. At least one year of network follow-up was available for all participants (i.e., network sampling ended in 2011), beginning in 2005 when network sampling was sufficiently dense for these analyses (Figure S3 in File S1). We defined TNS as the function of the total degree (**d**) of the node at baseline (**d** = 0 if no connections are inferred), conditioned on the network inferred at the time of each subject's baseline sequence (**N**). Specifically, *TNS(* ***d|N***)  =  Prob (*degree of a node in N<* ***d***), with the probability computed using the best-fitting parametric density for the network **N**. In other words, TNS of a node with degree **d** is the proportion of all network nodes with degrees less than **d**, estimated from the histogram smoothed by the fitted parametric distribution (Figure S3 in File S1). TNS could range between 0 and 1, with higher values representing nodes with unusually high connectivity [see Supplemental Methods]).

Next, we examined associations between the calculated TNS and baseline characteristics, including viral load (VL), CD4 count, risk behaviors (number or sex partners and unprotected anal intercourse [UAI]), stage of infection and demographics. We also investigated the relationship between putative transmissions, as measured by the accumulation of edges with assigned directions away from the participant between baseline and year 1. We also tested whether nodes with higher TNS were associated with a greater risk of participating in putative transmissions (i.e., out-edges) between baseline and year 1 (as calculation of TNS is independent of associated edge direction). The TNS values were neither shared with study participants, nor generated in real time to influence clinical decision-making.

### Evaluation if TNS Could Inform Prevention Strategies

**Figure 2:**
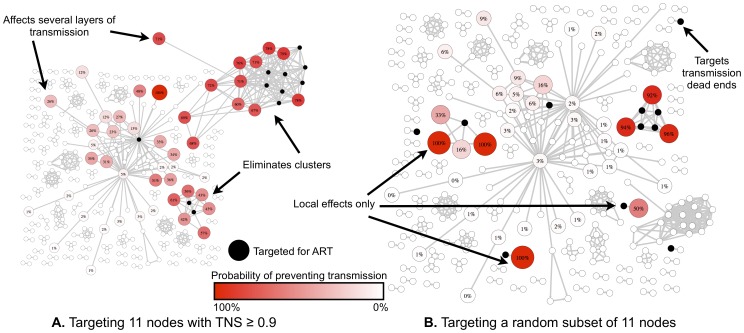
Simulations of ART provided either to those with the highest TNS or to a random subset of clustered individuals. Black nodes are those that are being treated (the assumption is that ART is 100% effective at stopping all secondary transmissions). Other nodes are colored according to how likely they are to be prevented from becoming infected assuming that we have removed the treated nodes from the infectious pool; they are also labeled by the rate at which they are expected to be effectively protected (dark red = very high probability of preventing transmission). These values are derived from simulating treatment where the randomness comes from the fact that should a node have N possible infectious connections, K of which are treated/removed due to treatment of other nodes, the node itself will NOT become infected with probability K/N. Targeting high TNS in panel A shows (i) The removal of an entire large cluster, where many nodes have high TNS (ii) prevented chains of transmission (i.e. even nodes that are not directly connected to the treatment subset have a high probability from being protected). Targeting the same number of random nodes in panel B shows: largely a very local effect and almost no chains being disrupted (with the exception of a cluster that is randomly chosen). Both panel A and panel B networks have the same topology, though the appearance is slightly different to allow labeling of specific nodes.

To assess how network information and the calculated TNS could possibly inform prevention strategies, we estimated the impact of the timing of ART within our cohort on the transmission dynamics. Specifically, we tested the level of network connectivity of participants between those who started ART early (i.e., within 12 weeks of EDI[13]) versus those who delayed ART >12 weeks from EDI. This analysis was based on the total degree network statistic developed by Wertheim et al [24]. The statistic is the difference between the total degree (defined as the sum of all the node degrees) of the groups. To decide whether this statistic is unusually low or unusually high, a null distribution is generated by permuting node labels, conditioned on the structure of the network. We also modeled the impact of targeted treatment with ART of a subset of individuals on preventing other infections using computer simulations (Figure 2 and Figure S4 in File S1). In this model we liberally assumed that ART would be 100% effective at stopping onward HIV transmission (see Supplementary Methods for details).

### Statistical Analyses

For TNS, a value of 0.75 or higher was defined as a “high” score. This represented the top quartile of TNS scores in our sample; all others were classified as “low”. The association between high TNS and patient characteristics, transmission risks, and clinical and epidemiological factors was tested using Wilcoxon-Mann-Whitney test for continuous characteristics, and Fisher's exact test for binary and categorical characteristics. Behavioral characteristics were examined independently. To ensure a linear relationship between each independent variable and the logit of the outcome, we log-transformed the viral load and the number of sex partners in the past year. Wilcoxon rank sum tests and Fisher's exact tests were used to compare participants with a new out-edge network connection (i.e. a putative transmission) to those without a new connection for continuous and categorical variables. A multiple logistic regression model was developed by considering variables that were statistically important (p<0.10) at the univariate level and then removing them using backward elimination (though the same results were obtained using forward and stepwise elimination). Benefits from adding covariates to the model were assessed with the likelihood ratio test. Goodness of fit of the final model was assessed by inspecting residuals and using the Hosmer-Lemeshow test. Confidence intervals on inferred network properties were obtained by drawing 1000 bootstrap replicates of the *pol* sequences, repeating network inference, and tabulating relevant statistics.

## Results

### Study Population

Between 1996 and 2011, the SDPIC screening program enrolled 648 HIV-1 infected individuals in the described network analysis, including 478 (73.8%) with recent HIV-1 infection and 170 of their HIV-1-infected sexual and social contacts. For the recently (i.e., acute and early) infected participants, the median time from the estimated date of infection (EDI) to presentation was 70 days (Table 1). Baseline participant characteristics were consistent with the epidemiology of HIV-1 in San Diego [12], [13]: most participants were male (96.0%), with a median age of 33 years, and men reporting sex with other men as the primary HIV-1 risk factor (Table 1). A total of 921 HIV-1 population *pol* sequences were isolated from 648 persons, nearly all being HIV-1 subtype B (98.5%) with sequences from 17.6% of participants harboring some drug resistance mutations (Figure S5 in File S1). A subset of 89 participants had multiple HIV-1 *pol* sequences generated during study follow-up with a median of two sequences per individual (range: 2–21) and a median duration of follow-up of 49 weeks (range: 1–413 weeks).

**Table 1:** Baseline characteristics of study participants.

Characteristics		SDPIC	Others	Total
Male, n = 630, n (%)		462 (96.7)	143 (94.1)	605 (96.0)
Age, n = 611, median (range)		33 (16–67)	35 (18–58)	33 (16–67)
Race/Ethnicity, n = 463	Non-Hispanic white, n (%)	155 (48.1)	63 (44.7)	218 (47.1)
	Non-Hispanic black, n (%)	14 (4.4)	22 (15.6)	36 (7.8)
	Hispanic, n (%)	122 (37.9)	42 (29.8)	164 (35.4)
	Other, n (%)	31 (9.6)	14 (9.9)	45 (9.7)
Risk Factors for HIV	MSM, n = 590, n (%)	444 (97.2)	125 (94.0)	569 (96.4)
	Heterosexual, n = 614, n (%)	29 (6.1)	16 (11.4)	45 (7.3)
	Injection drug use, n = 623, n (%)	17 (3.6)	12 (8.3)	29 (4.7)
Elapsed time	Days from HIV-1 infection to study entry, n = 478, median (range)	70 (7–170)	N/A*	N/A*
	Days from study entry to first *pol* sequence, n = 640, median (range)	0 (0–1462)	0 (0–1813)	0 (0–1813)
	Days from study entry to start or ART, n = 341, median (range)	72 (0–3718)	N/A*	N/A*
	Weeks from study entry to last visit, n = 634, median (range)	98.3 (0–667.7)	2.0 (0–293.2)	60.1 (0–667.7)
Laboratories (n = 616)	CD4 (cells/mm^3^), median (range)	500 (67–1380)	378 (7–2273)	484 (7–2273)
	Viral load (HIV–1 RNA log_10_ copies/mL), median (range)	5.0 (0–7.8)	4.1 (0–7.0)	4.9 (0–7.8)
	50–1500 copies/mL, n (%)	27 (5.7)	38 (27.5)	65 (10.6)
	1501–,000 copies/mL, n (%)	63 (13.2)	24 (17.4)	87 (14.1)
	10,001–100,000 copies/mL, n (%)	143 (29.9)	46 (33.3)	189 (30.7)
	>100,000 copies/mL, n (%)	245 (51.3)	30 (21.7)	275 (44.6)

SDPIC  =  San Diego Primary Infection Cohort, Others  =  non-SDPIC participants, MSM  =  men who have sex with men, ART  =  antiretroviral therapy.

*Date of infection and start of ART were not estimated for non-SDPIC participants.

### Transmission Network Characteristics

The HIV-1 *pol* sequences generated from each participant were used to infer the transmission network. Overall, the mean genetic distance between pairs of randomly selected baseline sequences was 5.83% (s.d. 1.46%), and pairwise distances below the threshold of 1.5% used to define a link between individuals, were rare overall (0.25%, Figure S1 in File S1). Using this 1.5% threshold, 339 individuals (52.3%, 95% CI: 333-392) were connected to at least one other study participant. Individuals were then divided into connected (i.e., clustered) and disconnected (i.e., singletons) nodes. Connected nodes (Figure 1) were arranged in 90 clusters (95% CI: 68–90), ranging in size from 2 to 62 individuals. It was possible to discern the direction of the putative HIV-1 transmission in 332 of the 540 connections (61.5%) by comparing the sampling date of the secondary partner and the EDI for the putative initial (i.e., transmitting) partner (Figure S2 in File S1). Overall, 18.5% (n = 29) of clustered participants had a new outbound connection within one year of enrollment. A total of 208 connections (38.5%) remained undirected because neither individual had an EDI (n = 29) or neither direction could be ruled out by examining EDI and sampling date information (n = 179). Interestingly, participants enrolled during acute and early infection were not significantly more likely to develop a new outbound edge within the first year of follow up than persons with established (i.e., chronic) infection (75.5% vs. 67.2%, p = 0.52). However, similar to previously described HIV-1 networks derived mostly from populations of men who have sex with men [6], [25], our network was best described by a preferential attachment model, indicating that new connections (i.e., putative transmissions) are more likely to form (or “attach”) to nodes that are already more highly connected (Figure S6 in File S1) [6].

### High TNS at Baseline Was Associated with Future Connections

Among 339 clustered participants, 157 were identified after 2004 and had TNS determined using only the information available at the time of study enrollment. The top quartile of the TNS distribution was designated as ‘high’ (TNS >0.75, n = 33) and all others as ‘low’ (n = 124). Participants with a high TNS were significantly more likely to have a putative transmission event within the first year, defined as one or more acquired outbound network connections in the first year (44.8% vs. 15.6%, p<0.01). The association between TNS and predicted risk of transmission was robust with regard to the cutoff chosen to determine “high” TNS (p<0.02 for TNS range in 0.70-0.95 [i.e., those in the top 25^th^ percentile]). Even as the network grew over time, the majority of high TNS nodes retained their unusually high connectivity.

### Clinical Correlates of Transmission

Participants with a higher baseline VL (median of 5.2 vs. 4.7 HIV RNA log_10_ copies/ml, p<0.01), and those with more sex partners at baseline (median of 3 vs. 1.5 partners, p = 0.03), were also significantly more likely to have a putative transmission event within the year of enrollment (Table 2). White participants (29.3%) were significantly more likely than Hispanics (11.1%) or participants of other race(s) (5.9%) to experience a putative transmission event (p = 0.02). There were no significant associations between baseline CD4 count (p = 0.53), insertive (p = 0.50) or receptive UAI (p = 0.34), age (p = 0.79), or stage of infection (p = 0.50) and putative transmission events. Baseline VL and high TNS were not significantly correlated in univariate analysis (p = 0.27), but in a multivariable analysis, number of unique sex partners, VL and high TNS (>0.75) at baseline were independently correlated with predicted risk of HIV transmission within the first year after presentation (p = 0.030, p = 0.003 and p = 0.005, respectively). Adding TNS to a logistic regression model of new network connections with VL as an explanatory variable contributed significantly to the model (p<0.001). There were also significant associations between TNS and baseline number of unique sexual partners (≥1) in the past month (p = 0.014) and insertive (p = 0.0455), but not receptive (p = 0.733) UAI.

**Table 2:** Clinical correlates of HIV transmission.

Baseline parameter		Putative transmission*		Analysis	
		Yes	No	Univariate	Multivariate
Plasma VL (median log_10_ RNA copies/mL)		5.2	4.7	p<0.01	OR = 2.0, P<0.01
CD4 count (median cells/µl)		382	469	p = 0.53	
Number of sex partners (median previous month)		3.0	1.5	p = 0.03	OR = 1.8†, P = 0.03
UAI, receptive	Any	76.0%	64.8%	p = 0.34	
	None	24.0%	35.2%		
UAI, insertive	Any	69.2%	60.4%	p = 0.50	
	None	30.8%	39.6%		
Race	White	75.9%	50.4%	p = 0.02	
	Hispanic	20.7%	28.1%		
	Other	3.5%	21.5%		
Stage of infection	Acute/Early	72.1%	64.1%	p = 0.52	
	Established	27.6%	35.9%		
TNS (>0.75)		44.8%	15.6%	<0.01	OR = 4.0, P<0.01

UAI  =  unprotected anal intercourse, VL  =  viral load.

*Defined as ≥1 acquired outbound network connection(s) in the first year after incident HIV infection.

† The log-transformed number of sex partners was used in the regression model, so OR = 1.8 corresponds to the odds of transmission for subjects with 1 sex partner compared to subjects with 0 sex partners (OR = 1.4 when comparing 2 to 1 sex partners).

### Evaluation of robustness to network inference error

TNS inferred from an incompletely sampled molecular network was a good predictor of the TNS in the unobserved larger transmission network, implying that despite the limitations of our approach (see Discussion), molecular networks likely retain key qualitative properties of actual transmission networks. Based on 100 simulations of transmission dynamics and sequence evolution of 5,000 HIV-1 pol sequences, followed by a subsampling of 648 sequences, network inference and TNS calculation, we found that in all 100 cases there was significant (p<0.05, Kendall rank correlation test) correlation between the true and inferred TNS. Furthermore, the median positive predictive value for high TNS (top quartile) based on molecular network data was 0.66.

### ART Analyses

To further understand how network information could be used for prevention efforts, we investigated if the preventative efforts of early initiation of ART [26] could be observed in the sampled network. A total of 177 out of 339 (52.2%) clustered participants initiated ART at a median of 168 days from study entry (i.e., baseline). Retrospective analysis using a network statistic developed by Wertheim et al. [24] showed that ART initiation within 12 weeks of EDI (n = 64) resulted in significantly less putative transmission (i.e. fewer out- and undirected- connections) than when ART was started later (p<0.05). We also evaluated if the network could possibly inform targeting of prevention interventions, and found through simulation that ART given to the 11 individuals with the highest TNS (≥0.90) and assumed to be 100% effective at preventing transmission, showed a greater probability of reducing HIV-1 transmissions compared to ART provided to the same number of randomly selected individuals in the network (Figure 2 and Table 3). This observation remained in 91% of simulated treatment scenarios to those with TNS ≥0.90. We also investigated whether selecting a subset for immediate treatment based on baseline demographics and reported risk behaviors would yield prevention improvements comparable to those achieved by TNS. Such simulations suggested that targeting ART for individuals based on the number of sex partners in the last month, whether or not they reported always having UAI in the last month or whether or not they had another sexually transmitted infection (STI), did not provide a measurable improvement over a randomly selected subset of nodes (probability of improving on a random treatment >0.5 in all cases).

**Table 3:** Simulations of targeted vs. random ART intervention.

TNS threshold	Individuals treated	TNS benefits			
		TNS-targeted	Randomly targeted*	Prevention yield improvement†	Probability that TNS is more efficient than random§
0.95	8	16 (11–18)	7 (5–10)	2.3	96%
0.9	11	17 (13–21)	10 (8–13)	1.7	91%
0.85	21	22 (18–26)	18 (15–22)	1.22	71%
0.8	30	23 (19–27)	24 (20–28)	0.89	40%

*Aggregated over 1000 random subsets of treated nodes.

† The ratio of the median number of prevented infections between TNS and randomly delivered ART.

§ The proportion of randomly targeted ART interventions that prevented fewer infections than TNS-targeted ART.

## Discussion

Although HIV-1 network structure and transmission dynamics have characterized temporal trends in identified HIV-1 cases [6], [27], these studies have rarely, except in Wertheim et al. [24], been used to predict or simulate future HIV-1 infections. By combining methods from classical and molecular epidemiology, we were able to infer and characterize the local transmission network. Specifically, we inferred the HIV-1 transmission network in San Diego, California using HIV-1 sequence data generated during routine drug resistance testing from a well-characterized cohort of recently infected individuals and their sexual and social contacts. We then evaluated if network characteristics could predict future transmission patterns.

**Figure 3:**
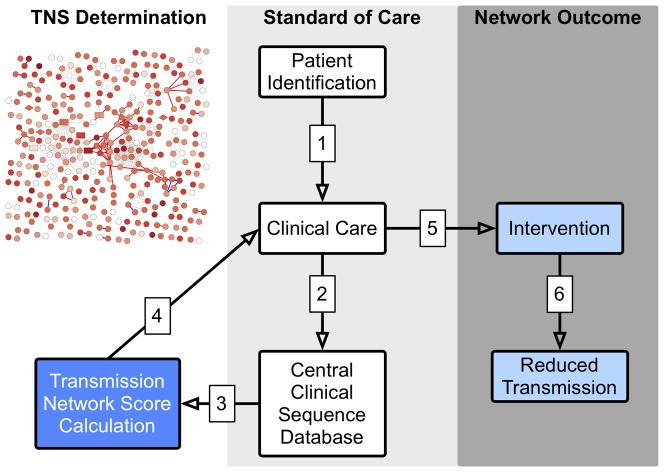
Schematic of TNS Clinical Application and Outcomes. The schematic illustrates in a step-by-step fashion (numbers 1-6), the application of TNS to clinical care and potential outcomes. The standard of clinical care for newly HIV diagnosed persons (1) includes baseline HIV *pol* sequence evaluation (2) to screen for ART drug resistance. With development of appropriate privacy preserving methods, these same data could be evaluated to determine a TNS (3). Feedback of TNS with drug resistance results (4), including an interpretation and description of potential limitations, could inform clinical care decisions (5). The opportunity to focus prevention intervention resources to those at greatest risk of subsequent HIV transmission could result in more efficient and effective use of these limited resources. Generalized use of these data within a transmission network is expected to reduce HIV transmission (6) to a greater degree than delivery of these same interventions provided at random (i.e., guided by traditional metrics of risk for disease progression and behavioral risk).

As a proof of principle, local HIV-1 transmission network characteristics were used to derive a score (TNS) that estimated the risk of transmission, during the first year of HIV-1 infection, when transmission risk may be greatest [27], [28]. This objective score identified a subset of participants (TNS >0.75) who had a significantly greater predicted risk of HIV transmission within their first year of infection than those with lower TNS. As evidence that TNS reflected a biologic correlate of transmission risk, a positive and correlation was observed between baseline VL (and TNS) and likelihood of acquiring an outbound edge within the first year [29]–[31]. When TNS was incorporated into a multivariate model with VL, the prediction of transmission risk significantly improved, suggesting that VL and TNS are informed by independent transmission risk factors (e.g., per contact transmission risk and number of high risk contacts). Taken together, TNS provides a new method to estimating transmission risk within a network, and this method could likely be extended to infer regional transmission networks from the extensive archives of HIV sequence data stored in commercial databases. Since the TNS is derived only from information available at the time of enrollment, the score could be readily utilized in clinical practice (Figure 3), as patient level TNS results could be integrated into routine baseline genotype test reports providing a general transmission risk interpretation to the patient's healthcare provider and the patient.

We then evaluated if network data can be used to help target prevention strategies. First, we retrospectively observed that self-selected early initiation of ART was associated with a cumulative decrease in putative transmission in our network, as compared to delaying ART. We then used data from our local network to inform simulations, that demonstrated that targeting individuals with highest TNS (≥0.90) with highly effective prevention interventions (e.g., fully suppressive ART), we would expect to reduce local network transmissions more efficiently than with the same prevention intervention targeting individuals with the greatest number of recent sexual partners or STI (Table 3). While encouraging, one still must prove that such interventions can disrupt the *entire* network if they are to appreciably reduce the incidence rate in the at risk community. Based on these robust HIV-1 network transmission observations, we therefore propose a method to use the connectivity of individuals to guide targeted prevention interventions, like early ART.

There are limitations to the interpretation of these results. The inferred transmission network is incomplete and inaccurate, and the presence of a directed link between two individuals does not guarantee an HIV transmission event occurred; it simply reflects recent relatedness of the virus, possibly through a series of unobserved intermediaries. While the inferred network only proposes “putative” transmissions, a limitation of the convenience-based sampling methods used for these analyses is that they are inadequate to discern with certainty true transmission chains from clusters of epidemiologically unlinked persons infected by a common or intermediate source. In addition, individuals who were infected by partners outside the well-sampled area (e.g. infected in a different city) will likely be assigned a low TNS score even if they pose a high risk of onward transmission. Similarly, nodes characterized by high TNS values may also represent lower risk individuals who are genetically linked to unobserved (i.e., unsampled) high risk intermediaries. Our recent work [32] on large-scale network reconstruction, where hundreds of thousand sequences can be included in the analysis of local transmission networks suggests a possible solution to by conditioning the local network in the context of a global network. In addition, by conditioning on the network structure, using robust statistics, and using community level measures these unobserved connections can be mitigated[24]. Further, more sophisticated methods [19] are being developed to help better associate molecular and epidemiological links. Also, these results may not be generalizable to other networks. The efficiency of HIV-1 transmission per contact (influenced by sexual behavior, VL, STI, etc. [33]) may vary by geographic region, thus optimal prevention interventions strategies may depend upon a thorough understanding of local transmission dynamics. Finally, there remains concerns about the potential loss of privacy related to disclosure of putative transmission between two or more individuals [34], even though there are significant limitations in proving direct HIV transmission links [35], [36]. Nevertheless, with appropriate privacy protection protocols, it is reasonable to consider using HIV transmission network data to develop prevention intervention strategies (Figure 3).

When adequately sampled, HIV-1 sequence analysis can help characterize local HIV epidemics. This network based study in San Diego, California corroborated previous findings that higher VL was associated with transmission risk [31] and that early ART decreased this risk [26]. This study went further to identify that network connections at baseline also predicted future transmission risk, and prevention efforts targeted to these individuals may be a better use of prevention resources than random implementation or targeting individuals with higher number of sexual partners or recently diagnosed with an STI. While traditional HIV partner services are critical to effective HIV prevention services, when combined with HIV molecular epidemiologic analyses, targeted use of available prevention and treatment resources to maximally limit HIV transmission may significantly reduce network, and ultimately population, HIV incidence. Awareness of HIV-1 transmission network characteristics could also help local public health officials and clinicians to focus HIV-1 screening and prevention education messages for particular groups over time.

## Supporting Information

File S1 **This contains Figures S1–S6, Tables S1–S2, and Supplemental Methods.**
(ZIP)

## References

[1] Guan Y, Peiris JS, Zheng B, Poon LL, Chan KH, et al (2004) Molecular epidemiology of the novel coronavirus that causes severe acute respiratory syndrome. Lancet 363: 99–104. 14726162 10.1016/S0140-6736(03)15259-2 PMC7112497

[2] Rivas AL, Fasina FO, Hoogesteyn AL, Konah SN, Febles JL, et al (2012) Connecting network properties of rapidly disseminating epizoonotics. PLoS ONE 7: e39778. 22761900 10.1371/journal.pone.0039778 PMC3382573

[3] Adams J, Moody J, Morris M (2013) Sex, drugs, and race: how behaviors differentially contribute to the sexually transmitted infection risk network structure. Am J Public Health 103: 322–329. 23237162 10.2105/AJPH.2012.300908 PMC3558752

[4] Lee S, Rocha LE, Liljeros F, Holme P (2012) Exploiting temporal network structures of human interaction to effectively immunize populations. PLoS One 7: e36439. 22586472 10.1371/journal.pone.0036439 PMC3346842

[5] Althaus CL, Heijne JC, Herzog SA, Roellin A, Low N (2012) Individual and population level effects of partner notification for Chlamydia trachomatis. PLoS One 7: e51438. 23251534 10.1371/journal.pone.0051438 PMC3520891

[6] Leigh Brown AJ, Lycett SJ, Weinert L, Hughes GJ, Fearnhill E, et al (2011) Transmission network parameters estimated from HIV sequences for a nationwide epidemic. J Infect Dis 204: 1463–1469. 21921202 10.1093/infdis/jir550 PMC3182313

[7] Gandhi NR, Weissman D, Moodley P, Ramathal M, Elson I, et al (2013) Nosocomial transmission of extensively drug-resistant tuberculosis in a rural hospital in South Africa. J Infect Dis 207: 9–17. 23166374 10.1093/infdis/jis631 PMC3523793

[8] Dennis AM, Hue S, Hurt CB, Napravnik S, Sebastian J, et al (2012) Phylogenetic insights into HIV transmission in North Carolina. Aids 26: 1813–22. 22739398 10.1097/QAD.0b013e3283573244 PMC3566771

[9] Hemelaar J, Gouws E, Ghys PD, Osmanov S (2011) Global trends in molecular epidemiology of HIV-1 during 2000–2007. AIDS 25: 679–689. 21297424 10.1097/QAD.0b013e328342ff93 PMC3755761

[10] Johnson VA, Calvez V, Gunthard HF, Paredes R, Pillay D, et al (2011) 2011 update of the drug resistance mutations in HIV-1. Top Antivir Med 19: 156–164. 22156218 PMC6148877

[11] Smith DM, May SJ, Tweeten S, Drumright L, Pacold ME, et al (2009) A public health model for the molecular surveillance of HIV transmission in San Diego, California. AIDS 23: 225–232. 19098493 10.1097/QAD.0b013e32831d2a81 PMC2644048

[12] Morris SR, Little SJ, Cunningham T, Garfein RS, Richman DD, et al (2010) Evaluation of an HIV nucleic acid testing program with automated Internet and voicemail systems to deliver results. Ann Intern Med 152: 778–785. 20547906 10.1059/0003-4819-152-12-201006150-00005 PMC2922925

[13] Le T, Wright EJ, Smith DM, He W, Catano G, et al (2013) Enhanced CD4+ T-cell recovery with earlier HIV-1 antiretroviral therapy. N Engl J Med 368: 218–230. 23323898 10.1056/NEJMoa1110187 PMC3657555

[14] Kosakovsky Pond SL, Frost SDW, Muse SV (2005) HyPhy: hypothesis testing using phylogenies. Bioinformatics 21: 676–679. 15509596 10.1093/bioinformatics/bti079

[15] Smith D, Delport W, Butler D, Little S, Richman D, et al (2010) Response to Comment on “The Origins of Sexually Transmitted HIV Among Men Who Have Sex with Men”. Science translational medicine 2: 501r1. 10.1126/scitranslmed.3001473 PMC3083242 21532938

[16] Brenner BG, Roger M, Routy JP, Moisi D, Ntemgwa M, et al (2007) High Rates of Forward Transmission Events after Acute/Early HIV-1 Infection. J Infect Dis 195: 951–959. 17330784 10.1086/512088

[17] Lewis F, Hughes GJ, Rambaut A, Pozniak A, Leigh Brown AJ (2008) Episodic sexual transmission of HIV revealed by molecular phylodynamics. PLoS Med 5: e50. 18351795 10.1371/journal.pmed.0050050 PMC2267814

[18] Volz EM, Koelle K, Bedford T (2013) Viral phylodynamics. PLoS Comput Biol 9: e1002947. 23555203 10.1371/journal.pcbi.1002947 PMC3605911

[19] Frost SD, Volz EM (2013) Modelling tree shape and structure in viral phylodynamics. Philos Trans R Soc Lond B Biol Sci 368: 20120208. 23382430 10.1098/rstb.2012.0208 PMC3678332

[20] Helleringer S, Kohler HP (2007) Sexual network structure and the spread of HIV in Africa: evidence from Likoma Island, Malawi. AIDS 21: 2323–2332. 18090281 10.1097/QAD.0b013e328285df98

[21] Janssen RS, Satten GA, Stramer SL, Rawal BD, O'Brien TR, et al (1998) New testing strategy to detect early HIV-1 infection for use in incidence estimates and for clinical and prevention purposes. JAMA 280: 42–48. 9660362 10.1001/jama.280.1.42

[22] Kothe D, Byers RH, Caudill SP, Satten GA, Janssen RS, et al (2003) Performance characteristics of a new less sensitive HIV-1 enzyme immunoassay for use in estimating HIV seroincidence. J Acquir Immune Defic Syndr 33: 625–634. 12902808 10.1097/00126334-200308150-00012

[23] Kassanjee R, Welte A, McWalter TA, Keating SM, Vermeulen M, et al (2011) Seroconverting blood donors as a resource for characterising and optimising recent infection testing algorithms for incidence estimation. PLoS ONE 6: e20027. 21694760 10.1371/journal.pone.0020027 PMC3111407

[24] Wertheim JO, Kosakovsky Pond SL, Little SJ, De Gruttola V (2011) Using HIV transmission networks to investigate community effects in HIV prevention trials. PLoS ONE 6: e27775. 22114692 10.1371/journal.pone.0027775 PMC3218056

[25] Schneeberger A, Mercer CH, Gregson SA, Ferguson NM, Nyamukapa CA, et al (2004) Scale-free networks and sexually transmitted diseases: a description of observed patterns of sexual contacts in Britain and Zimbabwe. Sexually Transmitted Diseases 31: 380–387. 15167650 10.1097/00007435-200406000-00012

[26] Cohen MS, Chen YQ, McCauley M, Gamble T, Hosseinipour MC, et al (2011) Prevention of HIV-1 Infection with Early Antiretroviral Therapy. The New England Journal of Medicine 365: 493–505. 21767103 10.1056/NEJMoa1105243 PMC3200068

[27] Hughes GJ, Fearnhill E, Dunn D, Lycett SJ, Rambaut A, et al (2009) Molecular phylodynamics of the heterosexual HIV epidemic in the United Kingdom. PLoS Pathog 5: e1000590. 19779560 10.1371/journal.ppat.1000590 PMC2742734

[28] Wawer MJ, Gray RH, Sewankambo NK, Serwadda D, Li X, et al (2005) Rates of HIV-1 Transmission per Coital Act, by Stage of HIV-1 Infection, in Rakai, Uganda. J Infect Dis 191: 1403–1409. 15809897 10.1086/429411

[29] Boily MC, Baggaley RF, Wang L, Masse B, White RG, et al (2009) Heterosexual risk of HIV-1 infection per sexual act: systematic review and meta-analysis of observational studies. Lancet Infect Dis 9: 118–129. 19179227 10.1016/S1473-3099(09)70021-0 PMC4467783

[30] Pilcher CD, Joaki G, Hoffman IF, Martinson FE, Mapanje C, et al (2007) Amplified transmission of HIV-1: comparison of HIV-1 concentrations in semen and blood during acute and chronic infection. Aids 21: 1723–1730. 17690570 10.1097/QAD.0b013e3281532c82 PMC2673564

[31] Quinn TC, Wawer MJ, Sewankambo N, Serwadda D, Li C, et al (2000) Viral load and heterosexual transmission of human immunodeficiency virus type 1. Rakai Project Study Group. N Engl J Med 342: 921–929. 10738050 10.1056/NEJM200003303421303

[32] Wertheim JO, Leigh Brown AJ, Hepler NL, Mehta SR, Richman DD, et al (2014) The global transmission network of HIV-1. J Infect Dis 209: 304–13. 24151309 10.1093/infdis/jit524 PMC3873788

[33] Beyrer C, Baral SD, van Griensven F, Goodreau SM, Chariyalertsak S, et al (2012) Global epidemiology of HIV infection in men who have sex with men. Lancet 380: 367–377. 22819660 10.1016/S0140-6736(12)60821-6 PMC3805037

[34] Rodriguez K, Castor D, Mah TL, Cook SH, Auguiste LM, et al (2013) Participation in research involving novel sampling and study designs to identify acute HIV-1 infection among minority men who have sex with men. AIDS Care 25: 828–34. 23305688 10.1080/09540121.2012.748164 PMC3688680

[35] Armstrong MP, Rushton G, Zimmerman DL (1999) Geographically masking health data to preserve confidentiality. Stat Med 18: 497–525. 10209808 10.1002/(sici)1097-0258(19990315)18:5<497::aid-sim45>3.0.co;2-#

[36] Bernard EJ, Azad Y, Vandamme AM, Weait M, Geretti AM (2007) HIV forensics: pitfalls and acceptable standards in the use of phylogenetic analysis as evidence in criminal investigations of HIV transmission. HIV Med 8: 382–387. 17661846 10.1111/j.1468-1293.2007.00486.x

