# Supplementary material for: Using HIV Networks to Inform Real Time Prevention Interventions
Source: PLoS One. 2014 Jun 5;9(6):e98443. doi: 10.1371/journal.pone.0098443 (PMC4047027; doi:10.1371/journal.pone.0098443)
Supplement: File S1 — This contains Figures S1–S6, Tables S1–S2, and Supplemental Methods. (ZIP) [file pone.0098443.s001.zip › Supporting information All/Little_Supplement_05-06-14_Clean.docx]

**Title: Using HIV Networks to Inform Real Time Prevention Interventions**

**SUPPLEMENTAL MATERIALS**

This supplemental section has been provided by the authors to give readers additional information about their work.

Susan J. Little, MD^1^*, Sergei L. Kosakovsky Pond, PhD^1^, Christy M. Anderson^1^, Jason A. Young, PhD^1^, Joel O. Wertheim, PhD^1^, Sanjay R. Mehta, MD^1^, Susanne May, PhD^2^ and Davey M. Smith, MD, MAS^1^

**SUPPLEMENTAL METHODS**

**Network Properties, Transmission Network Score, and Prevention Strategies**

To understand the dynamics of network formation, we fitted several models (described previously([5](#_ENREF_5))) to the observed degree distribution (Figure S3) of the local network and selected the best-fitting alternative. We identified the best fitting of four possible distributions used previously for sexual and epidemiological network analyses (negative binomial, Yule, Pareto, and Waring distributions)([6](#_ENREF_6)), using Bayesian Information Criterion([5](#_ENREF_5)). To estimate the reliability of network properties, including number of clusters, cluster sizes, and the parameters of the fitted degree distribution, we simulated 1000 bootstrap replicates of the sequence data, inferred a transmission network from each replicate, and tabulated the bootstrap distributions of parameters of interest. The density of sampled nodes within the network was insufficient for this estimation prior to 2005 (Figure S3, inset plot). From 2005 onwards, the Waring distribution with very similar parameter values was consistently chosen as the best fitting model.

Connected (i.e., clustered) nodes are characterized within the transmission network by the number of connections (i.e., degrees) to other participants. As illustrated schematically in Figure S2, the degree of the central node is 7 in panel A and 10 in panel B. We defined TNS as the function of the total degree (**d**) of the node at baseline, conditioned on the network known at the time of subject’s enrollment (**N**). Specifically, $TNS(d\left| N)=Prob(degree of a node in N\leq d) \right.$ with the probability computed using the best-fitting parametric density for the network **N**. Thus, a TNS of 0.92 means that the degree of a particular node is in the top 8% of all individuals clustering in the existing network. An example of putative transmission events designated by accumulation of outbound edges are shown in Supplemental Figure S2, with the central node acquiring two additional out-edges (red edges) within one year of baseline sampling (2009 to 2010), which could represent transmission events.

Briefly, the simulation procedure takes an existing network structure, and recursively eliminates a subset of individuals whose infections may have been stopped by administering ART to the target set. At stage 1, all nodes targeted by ART are eliminated. A subsequent stages, a new node is eliminated stochastically if it could have been infected by an already eliminated node, with probability proportional to how many of its undirected and inbound edges are connected to the already eliminated node. The algorithm stops when the entire network has been traversed. For each target set of nodes, the simulation is repeated 1000 times, and the median number of prevented infections is reported. When targeting subsets of random nodes, we selected them to have the same distribution of years of sampling as the corresponding set of TNS-based nodes, in order to accommodate non-uniform rate of sampling over time.

**Evaluation of robustness to inference error**

Transmission networks inferred from molecular sequence data will both miss real transmission events and create false linkages. The former is intuitively obvious: incomplete population sampling and long intervals between transmission and sequence sampling lead to lost linkage information. The latter is caused most prominently by inherent transitivity of molecular networks: if A transmits to B who transmits to C, then it is quite likely that the corresponding sequences are all genetically linked, forming the A,B,C triangle instead of the A-B-C transmission chain. To gauge how well TNS estimated from molecular networks resembles TNS defined on the complete (unobserved) transmission network, we performed a limited simulation study (note that the area of modeling and simulating HIV transmission in a population is an area of active research, see([7](#_ENREF_7)). Briefly, the simulation proceeded as follows:

(a) Simulate a transmission network of size 5000, starting with 25 initial (epidemiologically unlinked) viral lineages, following the Waring model (80% preferential attachment combined with 20% of random connection); on average every 100^th^ infection was introduced from outside the current network (to model introduction from other areas).

(b) We simulated the evolution of HIV sequences along this network; initial 25 lineages and out-of-the-area transmission chains were seeded with randomly chosen epidemiologically unlinked (TN93 distance ≥ 2.5%) subtype B sequences from the Los Alamos National Laboratory HIV database. The sequences were simulated under strict molecular clock, assuming the mean rate of substitution of 0.0015/site/year (see ([7](#_ENREF_7))), using the general time reversible nucleotide substitution model with site-to-site rate variation modeled using a 3-bin general discrete distribution with parameters estimated from the alignment of all SDPIC sequences. Timing of infections within a transmission chain followed an exponential distribution with mean of 14 days between infections, and each transmission event infected a random number of individuals, following the Poisson distribution with mean one (to simulate occasional “bursts” of transmissions). We further assumed that a sequence was sampled from an individual some time following transmission; based on the data from SDPIC this delay followed a bimodal (Beta) distribution with mean of 5 years, but with approximately 10% of individuals sampled much earlier.

(c) We subsampled 648 sequences (the same number of individuals as in the study sample) from the simulated network and applied exactly the same TNS inference procedure as we did on real data. Because of the focused effort of the SDPIC to enroll partners of existing study participants, we further assumed that such a partner of any sampled individual was 10 times as likely to be recruited as a random HIV infected individual.

(d) We computed whether or not the TNS estimated from molecular data correlated (Kendall rank correlation) with the TNS computed from the full unobserved transmission network, and whether or not individuals predicted to have TNS in the top quartile based on molecular data, had TNS in the top quartile based on the complete network.

The parameters of the simulation were chosen both to reflect what is known about the epidemic in San Diego, and to confirm that the inferred molecular networks closely resembled the following four properties of the network inferred from SDPIC: the number of connected nodes, the number of edges, the characteristic exponent of the degree distribution, and the mean pairwise TN93 distance between sequences.

**Statistical Methods**

For sequence analysis we used two software tools developed for this and related projects. First, a parallelized C++ program (TN93) was written to take aligned POL sequences, and report all pairs that had TN93 distances below a user-specified threshold. The program and accompanying documentation can be accessed at <https://github.com/veg/TN93>. Second, a Python library (hivclustering) was written in order to process the links output by the TN93 program and associated clinical data, to construct, visualize, and analyze the resulting molecular transmission network. In addition, a script needed to calculate the TNS from these networks is provided. This library is available at <https://github.com/veg/HIVClustering>. Multiple quality control procedures, including BLAST searches against reference HIV databases, comparisons with laboratory strains, and accurate phylogenetic subtyping were applied to remove potential contaminant sequences([8](#_ENREF_8), [9](#_ENREF_9)). Sequence clustering due to sample mislabeling was excluded by examining personal identifying characteristics on sampled individuals when available (i.e., same address, name, email, phone number, date of birth, etc), and by inspecting high-resolution HLA genotypes on samples with clustered sequences([9](#_ENREF_9)). Measurement of the nucleotide genetic distances (the TN93 model([10](#_ENREF_10))) between all pairs of sequences in the cohort was used to infer the transmission network.

**RESULTS**

Of the 648 baseline sequences, 478 (73.8%) were collected from recently infected participants at a median EDI of 70 days (Table S1), and 114 (17.6%) were predicted to harbor resistance to at least one drug class (Table S2) Not surprisingly, given that two linked sequences are genetically similar by definition, and hence likely share mutations, drug resistance associated mutations (DRAMs) were more frequently clustered than expected by chance (Figure S5). The number of connected nodes that harbored DRAM for a specific drug class was significantly higher than expected by chance (permutation test, conditioned on the network structure, 1000 replicates): p=0.03 (PI), p < 0.001 (NRTI, NNRTI, or any drug class).

The inferred molecular linkage was robust with 539 of 540 (99.8%) edges in the inferred transmission network present in ≥50% of bootstrap replicate networks (median edge bootstrap support of >99%). Repeating the analysis using only the baseline sequences excluded 17 edges and 6 nodes from the network; i.e., inclusion of longitudinal sequences added sampling depth as measured by greater connectivity. The inferred degree distribution had a heavy tail (Figure S3), a hallmark of scale-free networks([11](#_ENREF_11), [12](#_ENREF_12)) (Figure S6), where highly connected nodes are rare, but occur much more frequently compared to random networks.

We chose TN93 as a trade-off between computational speed and biological realism. Because TN93 distances can be evaluated directly from counts of nucleotide differences between sequences (and it is the most general nucleotide substitution model to have this property), it is possible to rapidly compute pairwise distances between tens of thousands of sequences. Because we focus on the estimation of short nucleotide distances, many known issues applicable for more divergent sequences (e.g. site-to-site rate variation) can be neglected. Indeed, TN93 distances less than 0.02 (2%) were essentially indistinguishable (linear correlation R^2^=0.99, p < 10^-16^) from those computed under much more general (and orders of magnitude slower) codon-based substitution models (results not shown).

Given the importance of transmission clusters in sustaining the rate of epidemic spread([12](#_ENREF_12), [13](#_ENREF_13)) and the potential public health benefit, it is essential to recognize the significant limitations related to proving HIV transmission associated with the clinical application of this information First, even in cases of near genetic sequence identity, sequence similarity alone cannot prove transmission([14](#_ENREF_14)). Second, without the necessary behavioral risk information and appropriately timed sample collection, there is no way to demonstrate HIV transmission as an outcome measure, even to validate the currently proposed TNS. Secure privacy preserving protocols can be developed to anonymize and encrypt these data with limits to both the amount and distribution of personal health information collected([15](#_ENREF_15)).

SUPPORTING INFORMATION: TABLES

Table S1. Summary of HIV Staging and Estimated Date of Infection (EDI) Algorithm

| **Stage** | **EDI Class*** | **N (%)** | **EIA/RT** | **LS-EIA** | **WB** | **RNA** | **Median (days)** | **IQR (days)** |
| --- | --- | --- | --- | --- | --- | --- | --- | --- |
| Acute | A-1.0 | 28 (17.8) | Neg. | - | Neg. | Pos. | 10 | (10 - 10) |
| Acute | A-2.0 | 4 (2.5) | Neg. | - | Ind. | Pos. | 15 | (8.5 – 19) |
| Early | A-3.0/3.1 | 6 (3.8) | Pos. | - | Pos.^†^ | Pos. | 34 | (30 – 88) |
| Early | E-1.0A | 44 (28.0) | Pos. | Present^§^ | Pos. | - | 70 | (70 – 70) |
| Early | E-1.0B/C | 12 (7.6) | Pos. | Present^‡^ | Pos. | - | 133 | (130 – 133) |
| Early | E-2.0 | 9 (5.7) | Pos. | - | Pos.^#^ | - | 100 | (39 – 112) |
| Chronic | E-3.0 | 54 (34.4) | Pos. | - | Pos.^Δ^ | - | - | - |

Class=PIRC stage of infection, EIA=enzyme immunoassay, RT=rapid test, LS-EIA=less sensitive EIA, WB=Western blot, IQR=interquartile range, Ind=indeterminate

*As defined in (1)

^†^ Within 30 days after a negative EIA or WB

^§^ LS-EIA with a result consistent with infection <70 days

^‡^ LS-EIA with a result consistent with infection between 70 and 170 days

^#^ Within 365 days after a negative EIA or WB (midpoint between first pos/last neg test)

^Δ^ Without a documented negative EIA or WB in previous 365 days, EDI estimated ≥180 days (2-4)

Table S2. Baseline Sequence Characteristics

| No. of Baseline Sequences | | 648 |
| --- | --- | --- |
| Variable | | No. Sequences (%) |
| Subtype | B | 638 (98.5) |
|  | Other | 10 (1.5) |
| Predicted Drug Resistance* | No resistance | 534 (82.4) |
|  | NRTI^†^ | 42 (6.5) |
|  | NNRTI^§^ | 77 (11.9) |
|  | PI^‡^ | 30 (4.6) |
|  | Only 1 drug class | 88 (13.6) |
|  | Any 2 drug classes | 17 (2.6) |
|  | All 3 drug classes | 9 (1.4) |
| Collection Date | 1996-2000 | 90 (13.9) |
|  | 2001-2005 | 280 (43.2) |
|  | 2006-2012 | 278 (42.9) |

*Drug resistance predicted when mutation confer a Stanford score ≥30

^†^ Nucleos(t)ide reverse transcriptase inhibitor

^§^ Non-nucleoside reverse transcriptase inhibitor

^‡^ Protease inhibitor

**SUPPORTING INFORMATION LEGENDS**

**Figure S1. Pairwise Genetic Distance (frequency vs. distance) in the SD PIC.**

The frequency distribution of pairwise nucleotide distance for the 921 sequences collected from 648 unique individuals is illustrated with a mean genetic distance at 5.8%. On the far left, there is a very small tail, highlighted in the inset plot, representing individuals with pairwise distances of ≤1.5% or potential transmissions.

**Figure S2. Schematic transmission dynamics used to generate Transmission Network Score.**

Directed edges (black and red) are oriented based on estimated duration of infection used to assign direction of transmission. Putative transmission direction is uncertain for undirected edges (grey). Each node is an HIV infected individual, and the number of edges (i.e., degrees) indicates that the genetic distance between the corresponding pair of *pol* sequences is ≤D. Singleton (i.e., unconnected) nodes are not linked to other nodes in the network. Panel A. Six directed edges between persons linked to central node are evident in 2009 (red outward edges indicate putative transmissions). Panel B. Evaluation of the central node sequence again within the context of the 2010 network shows the appearance of two new outward edges (red) consistent with potential *new* interval secondary transmissions. Connections between the central node and other nodes in the cluster remained stable.

**Figure S3. The degree distribution for clustered nodes in the SD PIC inferred transmission network**.

Inset plot characterizes network sampling density between 2000 and 2011. Error bars show bootstrap 95% confidence intervals. The dotted line depicts the fitted Waring distribution of degree counts. The inset plot shows the evolution of the number of nodes and the total number of edges in the network over time (2000-2011). The number of nodes and edges increase proportionally until 2005, after which time the ratio of edges to nodes identified increases over time suggesting that the network is more densely sampled.

**Figure S4. Schematic illustration of simulating the network effect of targeted ART treatment**.

Examples of treatment simulation. In Panel A, nodes 1 and 2 have 83% probability of removal, because each could have been infected by the targeted node (0), but also by each other. When considering whether or not to remove node 1, we consider that the probability that it was infected by node 0 is 2/3 (directed edge) and that it was infected by node 2 is 1/3 (undirected edge). Compare this to Panel B, where either 0 or 2 are equally likely. Even if node 1 is not removed in during stage 1, but node 2 was, then node 1 can still be removed with probability 50% in stage 2. Panel C shows the diminishing probability of removing nodes in subsequent stages, as we traverse the graph further and further from the index node. Finally, Panel D illustrates how multiple treated nodes interact to increase the probability of removing nodes in their cluster (4 and 6). Note that is also possible for node 4 to be removed as a consequence of preventing the infection of node 6 (even though it is not directly connected to nodes on ART). This is because we do not wish assume that a transmission event necessitates a direct link in the network: rather that both nodes belong to the same cluster.

**Figure S5. Transmitted drug resistance associated with HIV transmission clusters.**

Baseline drug resistance as predicted by mutations that confer a Stanford score ≥30^7^. The majority (82.4%) of participants demonstrated no drug resistance mutations. Resistance to one or more Nucleos(t)ide reverse transcriptase inhibitor (NRTI), Non-nucleoside reverse transcriptase inhibitor (NNRTI), or Protease inhibitor (PI) was observed in 42 (6.5%), 77 (11.9%), and 30 (4.6%), respectively.

**Figure S6. Schematic illustration of a random network (right) and a scale-free network (left), both with the identical number of nodes and edges (contacts).**

In the scale-free network, new nodes are more likely to attach to more connected nodes, resulting in the formation of hubs (indicated by the red arrow). In the random network, new nodes attach at random without the formation of hubs. Scale-free networks are widespread in nature and exhibit the property of preferential attachment and “predictable imbalance”([16](#_ENREF_16)). (An obvious example of a scale free network is that of the Internet, where there are a few large websites that most other websites are linked to, e.g. Facebook and Google.)

References

1. Le T, Wright EJ, Smith DM, He W, Catano G, et al. (2013) Enhanced CD4+ T-cell recovery with earlier HIV-1 antiretroviral therapy. N Engl J Med 368(3): 218-30.

2. Janssen RS, Satten GA, Stramer SL, Rawal BD, O'Brien TR, et al. (1998) New testing strategy to detect early HIV-1 infection for use in incidence estimates and for clinical and prevention purposes. JAMA 280(1): 42-8.

3. Kassanjee R, Welte A, McWalter TA, Keating SM, Vermeulen M, et al. (2011) Seroconverting blood donors as a resource for characterising and optimising recent infection testing algorithms for incidence estimation. PLoS ONE 6(6): e20027.

4. Kothe D, Byers RH, Caudill SP, Satten GA, Janssen RS, et al. (2003) Performance characteristics of a new less sensitive HIV-1 enzyme immunoassay for use in estimating HIV seroincidence. J Acquir Immune Defic Syndr. 33(5): 625-34.

5. Brown AE, Gifford RJ, Clewley JP, Kucherer C, Masquelier B, et al. (2009) Phylogenetic reconstruction of transmission events from individuals with acute HIV infection: toward more-rigorous epidemiological definitions. J Infect Dis 199(3): 427-31.

6. Leigh Brown AJ, Lycett SJ, Weinert L, Hughes GJ, Fearnhill E, et al. (2011) Transmission network parameters estimated from HIV sequences for a nationwide epidemic. J Infect Dis 204(9): 1463-9.

7. Eaton JW, Johnson LF, Salomon JA, Barnighausen T, Bendavid E, et al. (2012) HIV Treatment as Prevention: Systematic Comparison of Mathematical Models of the Potential Impact of Antiretroviral Therapy on HIV Incidence in South Africa. PLoS Med 9(7): e1001245.

8. Kosakovsky Pond SL, Posada D, Stawiski E, Chappey C, Poon AF, et al. (2009) An evolutionary model-based algorithm for accurate phylogenetic breakpoint mapping and subtype prediction in HIV-1. PLoS computational biology. 5(11): e1000581.

9. Mehta SR, Kosakovsky Pond SL, Young JA, Richman D, Little S, Smith DM. (2012) Associations between phylogenetic clustering and HLA profile among HIV-infected individuals in San Diego, California. J Infect Dis. 205(10): 1529-33.

10. Tamura K, Nei M. (1993) Estimation of the number of nucleotide substitutions in the control region of mitochondrial DNA in humans and chimpanzees. Mol Biol Evol 10(3): 512-26.

11. Albert R, Barabasi AL. (2002) Statistical mechanics of complex networks. Reviews of Modern Physics 74: 47-97.

12. Barabasi AL, Albert R. (1999) Emergence of scaling in random networks. Science 286(5439): 509-12.

13. Betts BJ, Shafer RW. (2003) Algorithm specification interface for human immunodeficiency virus type 1 genotypic interpretation. J Clin Microbiol 41(6): 2792-4.

14. Bernard EJ, Azad Y, Vandamme AM, Weait M, Geretti AM. (2007) HIV forensics: pitfalls and acceptable standards in the use of phylogenetic analysis as evidence in criminal investigations of HIV transmission. HIV Med 8(6): 382-7.

15. Armstrong MP, Rushton G, Zimmerman DL. (1999) Geographically masking health data to preserve confidentiality. Stat Med 18(5): 497-525.

16. Juran JM. (1975) The Non-Pareto Principle; Mea Culpa. Quality Progress. American Society for Quality Control 8(5): 8-9.
