# Supplementary material for: Using HIV Networks to Inform Real Time Prevention Interventions
Source: PLoS One. 2014 Jun 5;9(6):e98443. doi: 10.1371/journal.pone.0098443 (PMC4047027; doi:10.1371/journal.pone.0098443)
Supplement: File S1 — This contains Figures S1–S6, Tables S1–S2, and Supplemental Methods. (ZIP) [file pone.0098443.s001.zip › Supporting information All/Table S1.docx]

Table S1. Summary of HIV Staging and Estimated Date of Infection (EDI) Algorithm

| **Stage** | **EDI Class*** | **N (%)** | **EIA/RT** | **LS-EIA** | **WB** | **RNA** | **Median (days)** | **IQR (days)** |
| --- | --- | --- | --- | --- | --- | --- | --- | --- |
| Acute | A-1.0 | 28 (17.8) | Neg. | - | Neg. | Pos. | 10 | (10 - 10) |
| Acute | A-2.0 | 4 (2.5) | Neg. | - | Ind. | Pos. | 15 | (8.5 – 19) |
| Early | A-3.0/3.1 | 6 (3.8) | Pos. | - | Pos.^†^ | Pos. | 34 | (30 – 88) |
| Early | E-1.0A | 44 (28.0) | Pos. | Present^§^ | Pos. | - | 70 | (70 – 70) |
| Early | E-1.0B/C | 12 (7.6) | Pos. | Present^‡^ | Pos. | - | 133 | (130 – 133) |
| Early | E-2.0 | 9 (5.7) | Pos. | - | Pos.^#^ | - | 100 | (39 – 112) |
| Chronic | E-3.0 | 54 (34.4) | Pos. | - | Pos.^Δ^ | - | - | - |

Class=PIRC stage of infection, EIA=enzyme immunoassay, RT=rapid test, LS-EIA=less sensitive EIA, WB=Western blot, IQR=interquartile range, Ind=indeterminate

*As defined in (1)

^†^ Within 30 days after a negative EIA or WB

^§^ LS-EIA with a result consistent with infection <70 days

^‡^ LS-EIA with a result consistent with infection between 70 and 170 days

^#^ Within 365 days after a negative EIA or WB (midpoint between first pos/last neg test)

^Δ^ Without a documented negative EIA or WB in previous 365 days, EDI estimated ≥180 days (2-4)
