# Supplementary material for: Using HIV Networks to Inform Real Time Prevention Interventions
Source: PLoS One. 2014 Jun 5;9(6):e98443. doi: 10.1371/journal.pone.0098443 (PMC4047027; doi:10.1371/journal.pone.0098443)
Supplement: File S1 — This contains Figures S1–S6, Tables S1–S2, and Supplemental Methods. (ZIP) [file pone.0098443.s001.zip › Supporting information All/Table S2.docx]

Table S2. Baseline Sequence Characteristics

| No. of Baseline Sequences | | 648 |
| --- | --- | --- |
| Variable | | No. Sequences (%) |
| Subtype | B | 638 (98.5) |
|  | Other | 10 (1.5) |
| Predicted Drug Resistance* | No resistance | 534 (82.4) |
|  | NRTI^†^ | 42 (6.5) |
|  | NNRTI^§^ | 77 (11.9) |
|  | PI^‡^ | 30 (4.6) |
|  | Only 1 drug class | 88 (13.6) |
|  | Any 2 drug classes | 17 (2.6) |
|  | All 3 drug classes | 9 (1.4) |
| Collection Date | 1996-2000 | 90 (13.9) |
|  | 2001-2005 | 280 (43.2) |
|  | 2006-2012 | 278 (42.9) |

*Drug resistance predicted when mutation confer a Stanford score ≥30

^†^ Nucleos(t)ide reverse transcriptase inhibitor

^§^ Non-nucleoside reverse transcriptase inhibitor

^‡^ Protease inhibitor
