# Supplementary figures and images for: Using HIV Networks to Inform Real Time Prevention Interventions
Source: PLoS One. 2014 Jun 5;9(6):e98443. doi: 10.1371/journal.pone.0098443 (PMC4047027; doi:10.1371/journal.pone.0098443)

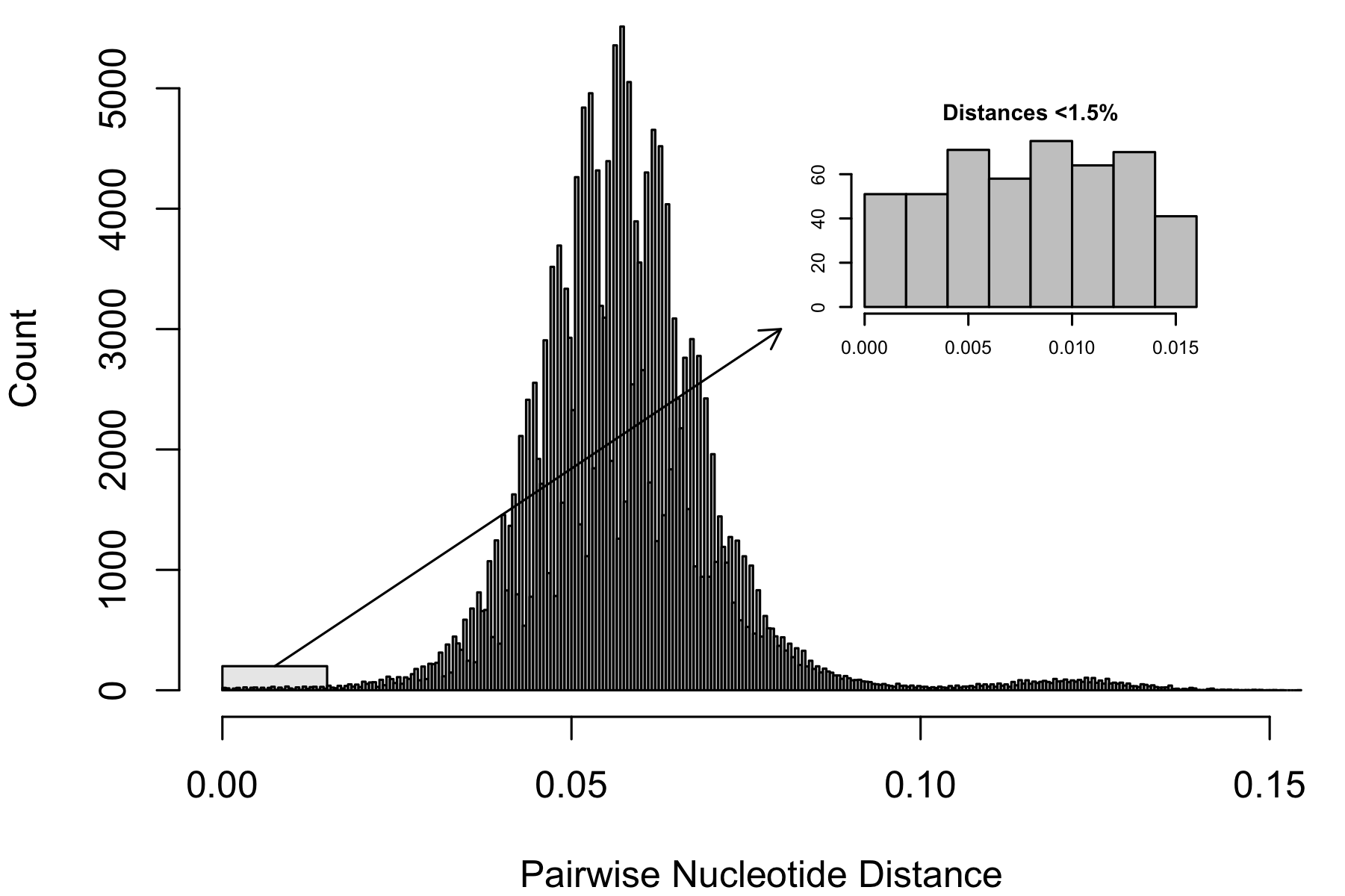

Supplement: File S1 — This contains Figures S1–S6, Tables S1–S2, and Supplemental Methods. (ZIP) [file pone.0098443.s001.zip › Supporting information All/Figure S1.tiff]

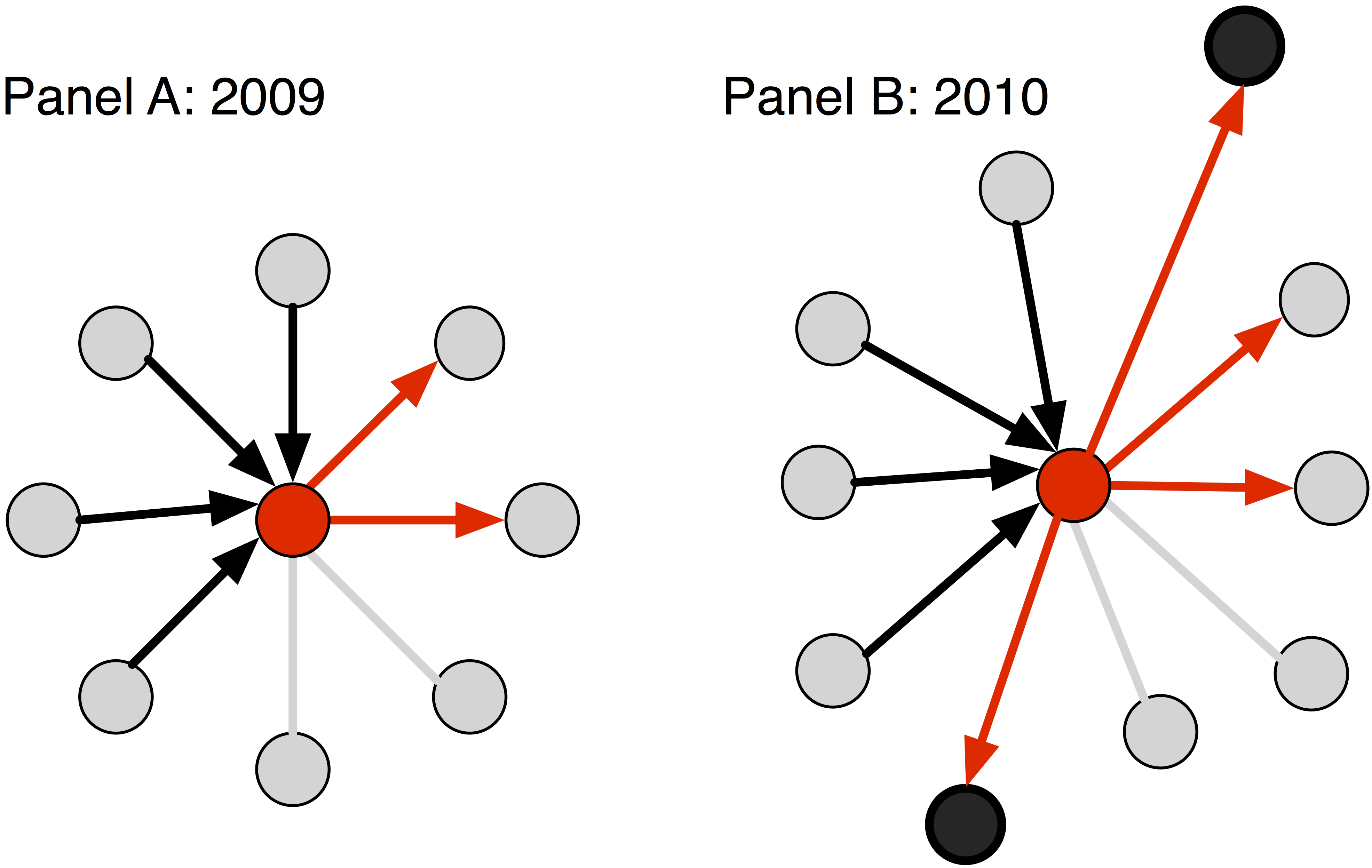

Supplement: File S1 — This contains Figures S1–S6, Tables S1–S2, and Supplemental Methods. (ZIP) [file pone.0098443.s001.zip › Supporting information All/Figure S2.tiff]

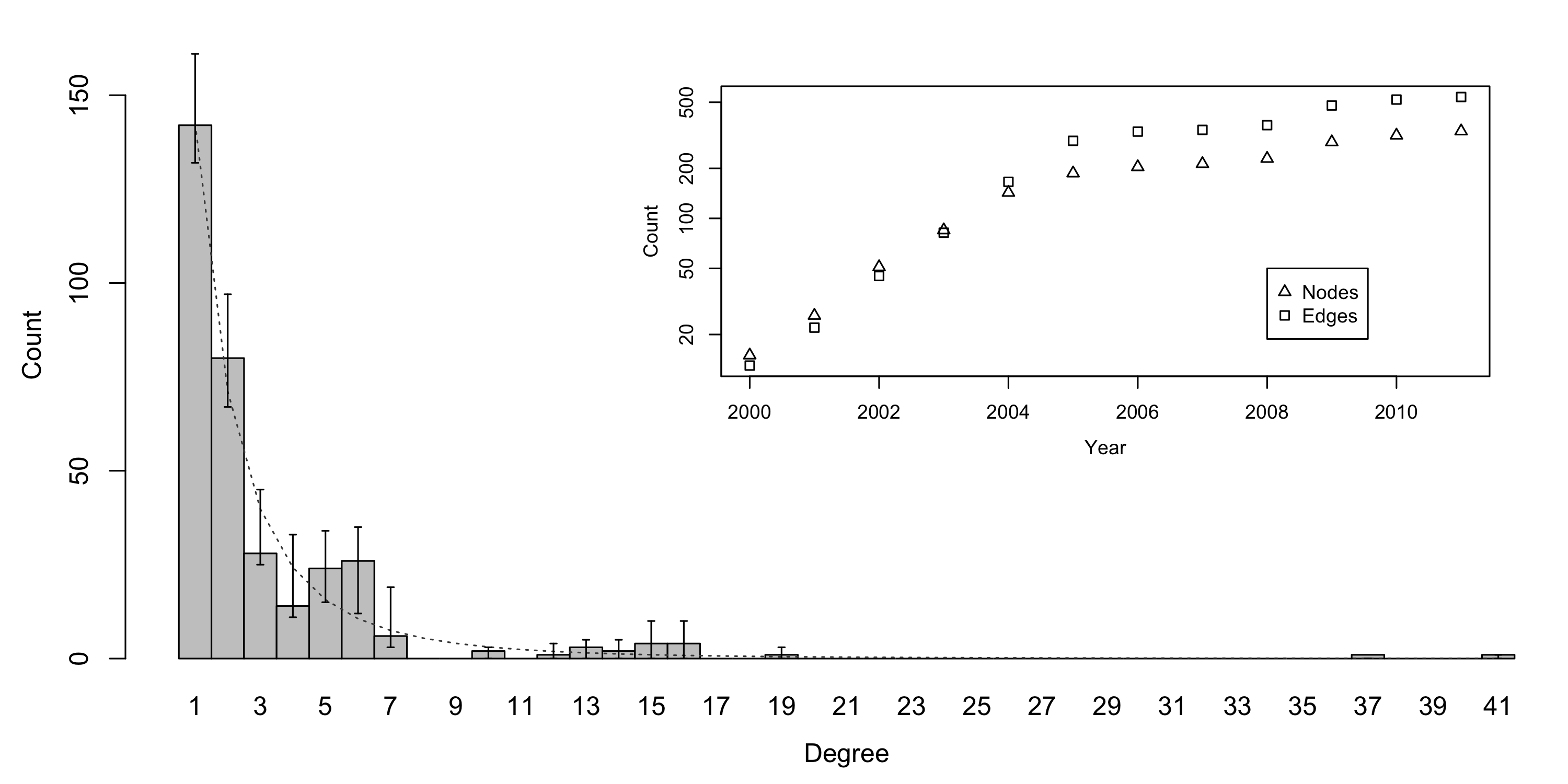

Supplement: File S1 — This contains Figures S1–S6, Tables S1–S2, and Supplemental Methods. (ZIP) [file pone.0098443.s001.zip › Supporting information All/Figure S3.tiff]

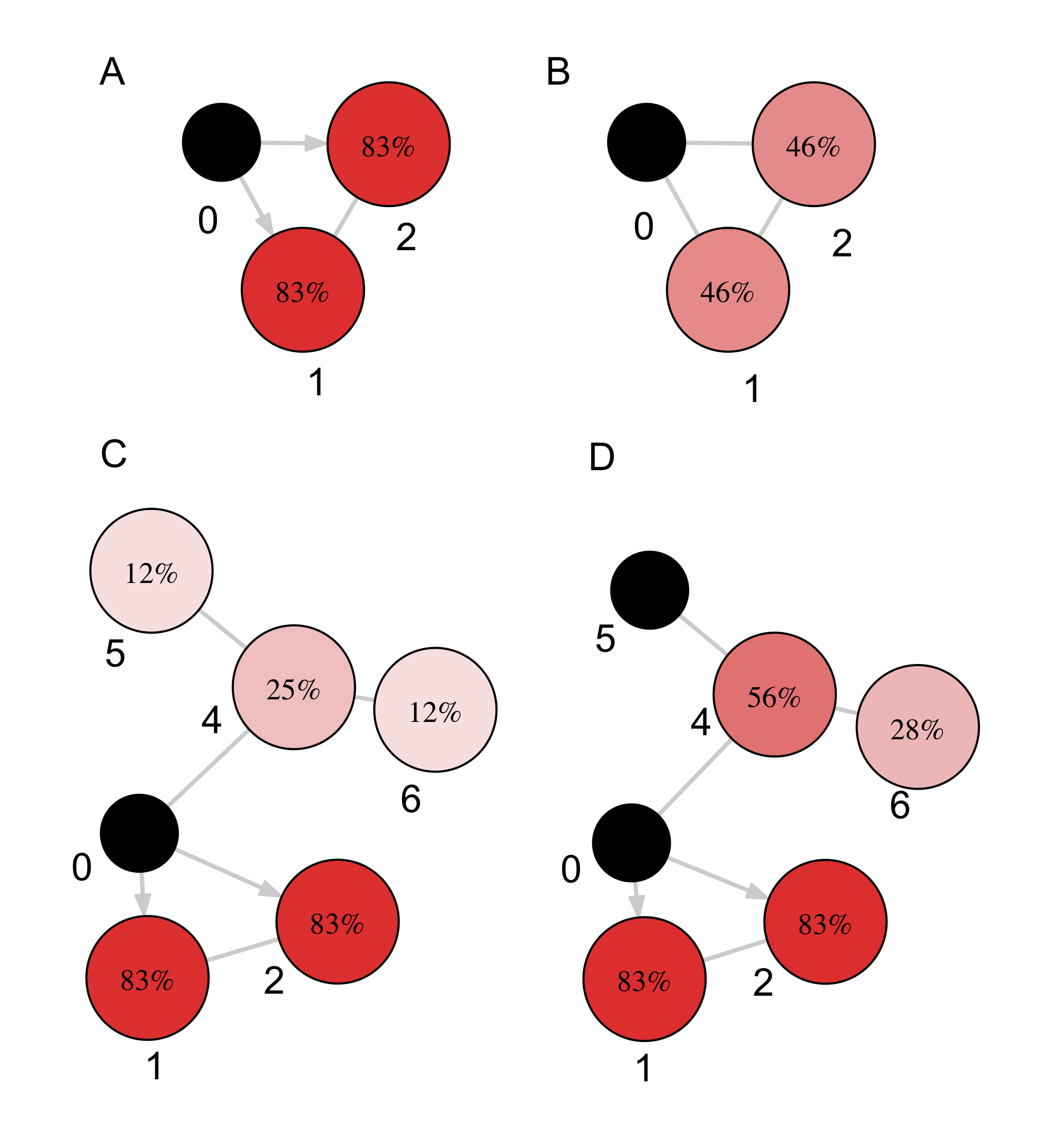

Supplement: File S1 — This contains Figures S1–S6, Tables S1–S2, and Supplemental Methods. (ZIP) [file pone.0098443.s001.zip › Supporting information All/Figure S4.tiff]

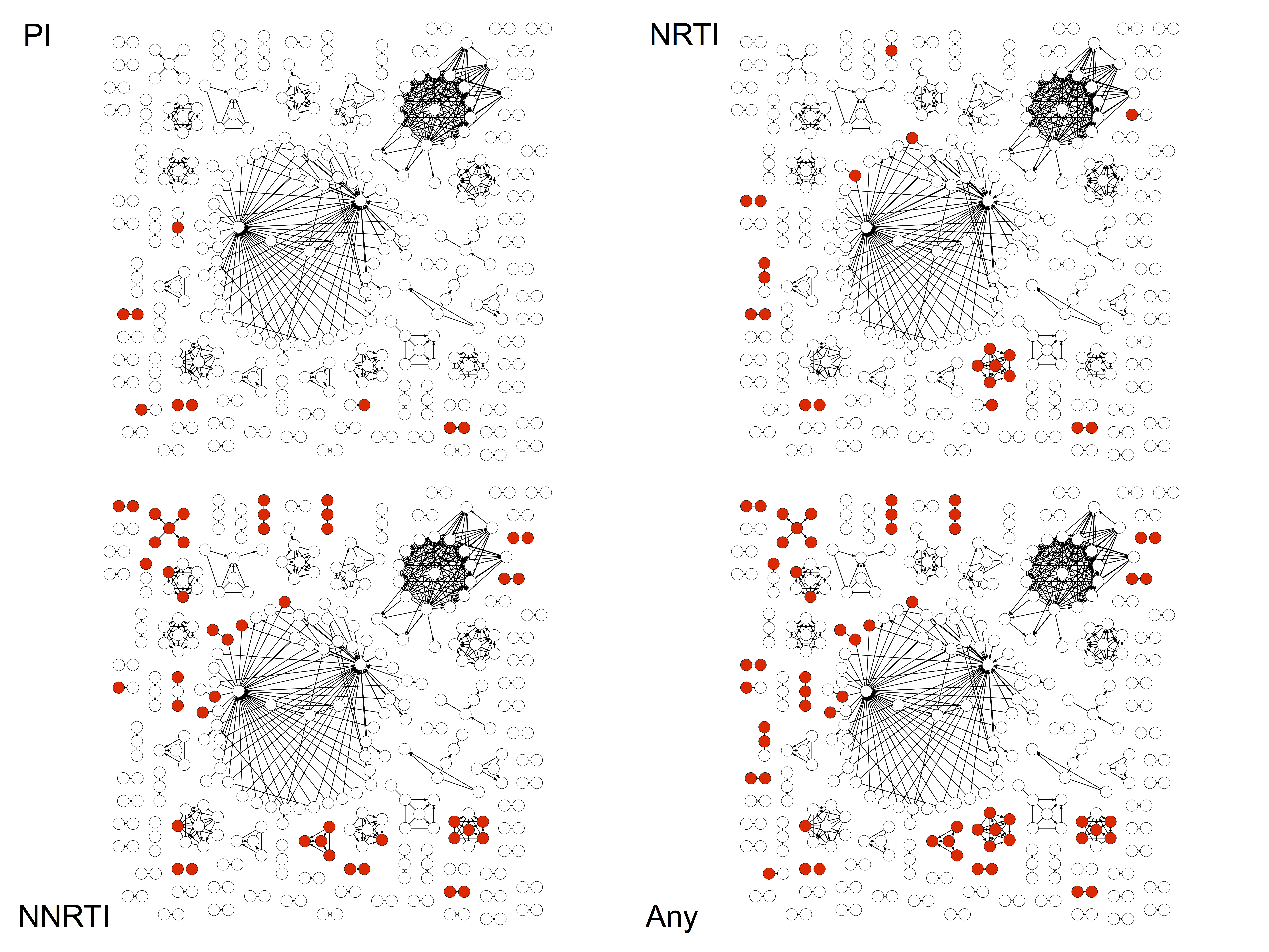

Supplement: File S1 — This contains Figures S1–S6, Tables S1–S2, and Supplemental Methods. (ZIP) [file pone.0098443.s001.zip › Supporting information All/Figure S5.tiff]

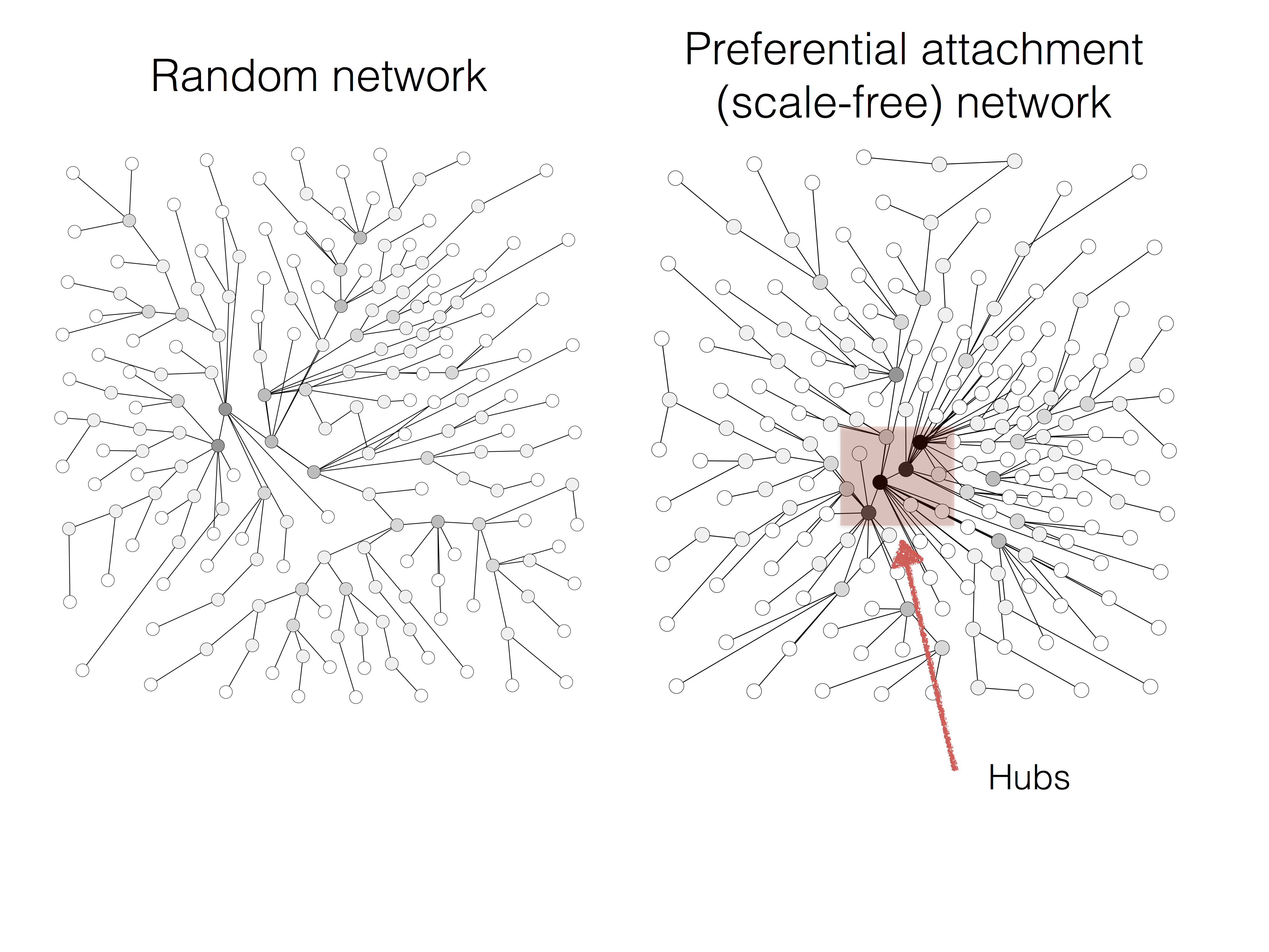

Supplement: File S1 — This contains Figures S1–S6, Tables S1–S2, and Supplemental Methods. (ZIP) [file pone.0098443.s001.zip › Supporting information All/Figure S6.tiff]
